# Transcriptional Profiling of Exosomes Derived from *Staphylococcus aureus*-Infected Bovine Mammary Epithelial Cell Line MAC-T by RNA-Seq Analysis

**DOI:** 10.1155/2021/8460355

**Published:** 2021-07-29

**Authors:** Yu Chen, Hongyuan Jing, Miaoyu Chen, Wan Liang, Jing Yang, Ganzhen Deng, Mengyao Guo

**Affiliations:** ^1^College of Veterinary Medicine, Northeast Agricultural University, Harbin 150030, China; ^2^College of Veterinary Medicine, Huazhong Agricultural University, Wuhan 430070, China

## Abstract

Mastitis is a common disease in the dairy industry that causes huge economic losses worldwide. Exosomes (carrying proteins, miRNA, lncRNA, etc.) play a vital role in the regulation of immune response. lncRNA can play a variety of regulatory roles by combining with protein, RNA, and DNA. The expression of mRNA and lncRNA in exosomes derived from bovine mammary epithelial cells infected by *S. aureus* is rarely understood. To explore this issue, RNA sequencing analysis was performed on exosomes derived from *S. aureus*-infected and noninfected MAC-T cells. Analysis of the sequencing results showed that there were 186 differentially expressed genes, 431 differentially expressed mRNAs and 19 differentially expressed lncRNAs in the exosomes derived from *S. aureus*-infected and noninfected MAC-T cells. By predicting lncRNA target genes, it was found that 19 differentially expressed lncRNAs all acted on multiple mRNAs in cis and trans. GO analysis revealed that differentially expressed genes and lncRNA target genes played significant roles in such metabolism (reactive oxygen species metabolic processes), transmembrane transport, cellular response to DNA damage stimulus, and response to cytokines. KEGG enrichment indicated that lncRNA target genes gathered in the TNF pathway, Notch pathway, MAPK pathway, NF-kappa B pathway, Hippo pathway, p53 pathway, reactive oxygen species metabolic processes, and longevity regulating pathway. In summary, all data indicated that differentially expressed gene, mRNA, and lncRNA in transcriptional profiling of exosomes participated in bacterial invasion and adhesion, oxidative stress, inflammation, and apoptosis-related signaling pathway. The data obtained in this study would provide valuable resource for understanding the lncRNA information in exosomes derived from dairy cow mammary epithelial cells and conduced to the study of *S. aureus* infection in dairy cow mammary glands.

## 1. Introduction

Mastitis is a common and widespread infectious disease in dairy farms all over the world, which is characterized by inflammation of the mammary glands, resulting in a decrease in milk production and quality [1–3]. There are many kinds of pathogens of mastitis, and *Staphylococcus aureus* (*S. aureus*) is one of the important pathogens in subclinical mastitis [4]. *S. aureus* often causes clinical or subclinical mastitis, and the intramammary infections are usually characterized as mild, chronic, or persistent [5].

Bovine mammary epithelial cells (BMEC) are not only essential for the synthesis of milk components but also the main line of defense against pathogen invasion [3, 6]. Milk contains a large number of exosomes, which are mainly derived from BMEC. The cargo carried by milk exosomes has been suggested to play a role in cell growth, development, immune regulation, and regulation [7]. Exosomes are tiny extracellular vesicles with a diameter ranging from 40 to 150 nm, which originate from the internal vesicles of multivesicles in almost all cell types [8]. Typical exosomes are surrounded by phospholipid membranes, which contain proteins, lipids, and nucleic acid molecules (including miRNA, cicrRNA, and lncRNA) [9]. With the deepening of the understanding of exosomes, exosomes are used as carriers to deliver drugs for targeted therapy. Because exosomes have been proven to tolerate the harsh environment in the intestine, they are absorbed by various cell types, cross biological barriers, and reach surrounding tissues [7]. Exosomes can also initiate a signal cascade of neighboring cells through the paracrine pathway. Therefore, it is very valuable to explore the RNA sequence in exosomes derived from MAC-T cells infected by *S. aureus*.

Long noncoding RNA (lncRNA) is a type of RNA that does not have the ability to encode proteins with more than 200 nucleotides in length [10]. LncRNA can bind to DNA, RNA, and proteins to play a role by mediating DNA methylation, mRNA degradation, chromatin remodeling, histone modification, and protein modification [10, 11]. There have been research evidences that lncRNA plays a key role in mastitis caused by *S. aureus*, *Escherichia coli*, *Mycoplasma bovis*, and other pathogens though regulating the expression of specific genes. LRRC75A-AS1 in bovine mammary epithelial cells and breast tissue was downregulated under inflammatory conditions, and knocking out LRRC75A-AS1 in MAC-T cell line inhibited the adhesion and invasion of *S. aureus* and attenuated the activation of the NF-*κ*B pathway [12]. LncRNA-TUB was the higher expression in the mammary epithelial cells of dairy cows that received proinflammatory stimulation, and knockout of lncRNA-TUB indicated that it played a key role in the morphology, proliferation, migration, and *β*-casein secretion of mammary epithelial cells [13].

Due to the spongy effect of exosomes, we speculated that the expression profile of lncRNA had changed in the exosomes derived from MAC-T cells infected with *S. aureus*. The purpose of this study was to determine the expression profile of lncRNA in exosomes derived from *S. aureus*-infected and noninfected MAC-T cells to provide new targets for the link between bovine mastitis caused by *S. aureus* and lncRNA for future research.

## 2. Methods

### 2.1. Cell Culture and Supernatant Collection

The bovine mammary epithelial cell line MAC-T was cultured in DME/F-12 (1 : 1) medium (Hyclone, USA) supplemented with 5 mmol/L L-glutamine (BioSharp, China), 5 *μ*g/mL insulin (BioSharp, China), 1 *μ*g/mL hydrocortisone (Sigma-Aldrich, USA), 100 U/mL penicillin/100 *μ*g/mL streptomycin (Invitrogen, USA), and 10% exosome-depleted fetal bovine serum (ExoDP-FBS; Gibco, USA) at 37°C with 5% CO2 incubator. *S. aureus* CVCC3051 strain, obtained from China Veterinary Culture Collection (CVCC), was grown in tryptone soy broth medium until midlog phase. After colony forming units (CFU) calculated, *S. aureus* was inactivated in hot water bath at 80°C for 30 minutes. When MAC-T cells were approximately 60-70% confluent in the T-75 cell flask, *S. aureus* was inoculated into the cell flask at a multiplicity of infection (MOI) of 100. After 24 hours of culture, the cell supernatant was collected for separation of exosomes.

### 2.2. Exosomes Isolation

Cell line exosomes were separated by ultracentrifugation as previously described [14]. The cell supernatant was sequentially subjected to 300×g, 10 min; 2000×g, 10 min; 10,000×g, 30 min in a large-capacity high-speed centrifuge (AVANTI J-26XPN, Beckman, USA). After collecting the supernatant, ultrahigh-speed centrifuge with SW32Ti rotor (optima XE-90, Beckman, USA) was hired to ultracentrifuge at 100,000×g for 70 min. Pellet was collected and resuspended in PBS and then performed ultracentrifugation again at 100,000×g for 70 min. Finally, the exosomal pellet was resuspended in PBS and collected for subsequent analysis.

### 2.3. Transmission Electron Microscopy

Exosomes were mixed with an equal volume of 4% paraformaldehyde. 100 *μ*L mixed liquid was sucked off and added dropwise to the copper net; then, phosphotungstic acid was added for negative staining. After drying, transmission electron microscope was used to observe exosomal morphology at 80 kV.

### 2.4. Particle Size Distribution

Exosomes obtained by ultracentrifugation was diluted by PBS to the optimal concentration for analysis. ZETASIZER Nanoparticle Tracking Analysis (NTA) instrument (Malvern, Worcestershire, England) was performed to analyze particle size of exosome. According to the Stokes-Einstein principle, dynamic light scattering detected Brownian motion of particles to generate trajectories and displacements to determine particle size distribution.

### 2.5. Flow Cytometry Analysis

Exosomes acquired by ultracentrifugation separation were incubated with CD63 (#557288, BD Bioscience) or CD81 (#555676, BD Bioscience), and exosomes without antibody incubation were used as a negative control. BD accuri C6 flow cytometer was used for sample detection according to the operating instructions and then analyzed with FlowJo software.

### 2.6. RNA Extraction and lncRNA Sequencing

Total RNA was extracted from exosomal samples using Trizol reagent (Invitrogen, Carlsbad, CA). Total RNA is used as an input material for constructing cDNA library. Mainly, exosomal trace RNA purification, cDNA synthesis through reverse transcription, end repair and adaptor, PCR amplification, and other steps build a library and then go through the library quality inspection and sequence on an Illumina HiSeq 4000 platform (Illumina Inc., San Diego, CA).

### 2.7. Sequence Data Quality Control

The original image file (BCL) obtained by sequencing was converted into raw data in FASTQ format after base recognition. Quality analysis on the original data was performed to assess whether it was suitable for bioinformatics analysis. Quality analysis mainly included sequencing quality and base composition analysis. Clean data was obtained by removing linker sequence and low quality sequence from raw data. The quality of clean data was then evaluated using FASTQC v0.10.1.

### 2.8. Reference Genome Alignment and Genome-Wide Read Distribution Map

Clean reads were obtained through filtering sequencing data by Trimmomatic (Version 3) and removing ribosomal RNA by Bowtie2 (v2.2.5). Bowtie2 (v2.2.5) was used to generate the index of the reference genome, and alignment of paired-end clean reads to the reference genome was performed with HISAT2 (v2.1.0). The assembly of mapped transcripts for each sample was performed with Scripture (beta2) and Cufflinks (v2.1.1) in a reference-based approach [15].

### 2.9. Differential Expression Analysis

Quantitative analysis of genes and transcripts was performed via htseq v0.11.2. DESeq2 (1.18.1) is used to determine differential expression in transcripts or gene expression data. It is determined that the transcripts or genes with *P* value < 0.05 are differentially expressed in biological replication. *Q* value < 0.05 and ∣log2 (fold change) | <1 are considered as thresholds for significant differential expression in abiotic replication.

### 2.10. Prediction of lncRNA-Gene Interactions

In order to predict the targets of lncRNAs in MAC-T cells and thus understand potential functions of lncRNAs, method as in previous research [15], firstly, we searched the 10 k/100 k coding gene upstream and downstream of lncRNA to find cis role lncRNA. Then, construction logarithm was modeled as log (*P*/(1 − *P*)) = *a* + *bx* + *cy*, where *a*, *b*, and *c* were model parameters, *y* represented the percentage of GC content in the sequence, and *P* represents lncRNA binding to a 10 kb long sequence odds ratio. Predict statistical significance by using Wald test on *Z* statistics (*P* value <0.05). Only lncRNAs with *P* value <0.05 and *z* score > 0 were annotated as trans-lncRNA.

### 2.11. Kyoto Encyclopedia of Genes and Genomes (KEGG) Biological Pathway Enrichment and Gene Ontology (GO) Functional Enrichment Analysis

The GOseq R package was used to analyze GO enrichment for differentially expressed genes or lncRNA target genes [15]. GO terms with *Q* values < 0.05 were considered significantly enriched by differentially expressed genes. The summary differential expression genes and lncRNA target genes in KEGG pathway enrichment were analyzed by KOBAS (v3.0) software.

### 2.12. RT-qPCR Validation

Total exosomal RNA samples from *S. aureus*-infected and noninfected MAC-T cells for the lncRNA-seq were analyzed by RT-qPCR. Then, cDNA was obtained by reverse transcriptase reagent according to the instruction manual. The qPCR was performed using the SYBR Green Plus Reagent Kit with the Light Cycler 96 instrument (Roche, Basel, Switzerland) following the instructions of the manufacturer. GAPDH was used as an internal reference gene. The primer sequences used in the study were listed in Table 1.

### 2.13. Statistical Analysis

IBM SPSS 20 was used to perform statistical analyses. The one-way analysis of variance (ANOVA) was performed to detect statistical differences in lncRNAs and mRNAs between *S. aureus*-infected and noninfected groups. *P* < 0.05 and *P* < 0.01 were considered as significant differences.

## 3. Results

### 3.1. Identification of Exosomes

**Figure 1:**
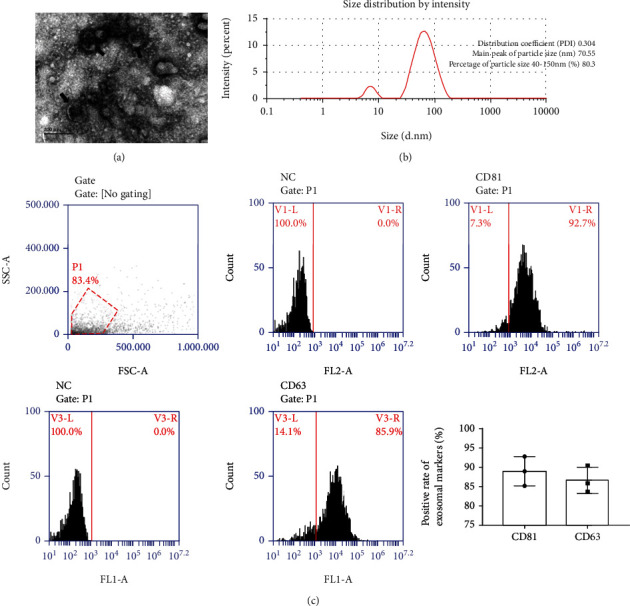
Identification of exosomes. (a) The morphology of exosomes under transmission electron microscope, scale bar = 200 nm; black arrows indicated exosomes. (b) Exosomal particle size analysis was measured by NTA instrument. (c) Exosomal characteristic markers CD81 and CD63 were detected by flow cytometry. NC was negative control; P1 was gate.

In order to directly observe the morphology of exosomes, we employed transmission electron microscopy to observe negatively stained exosomes samples. As shown in Figure 1(a), it had a very obvious single-layer membrane structure under the electron microscope, which appeared as a saucer-shaped or disc-shaped vesicle with one side recessed. NTA analysis was performed to further determine the size of exosomes. The main peak of the particle size of the exosomes we separated was 70.55 nm, and the 80.3% particle size was between 40 and 150 nm (Figure 1(b)). Flow cytometry was used to detect the surface characteristic markers of exosomes. The results revealed that the positive rates of CD81 and CD63 were 92.7% and 85.9%, respectively (Figure 1(c)).

### 3.2. Sequencing Data Statistics of Exosomes Derived from MAC-T Cells

**Figure 2:**
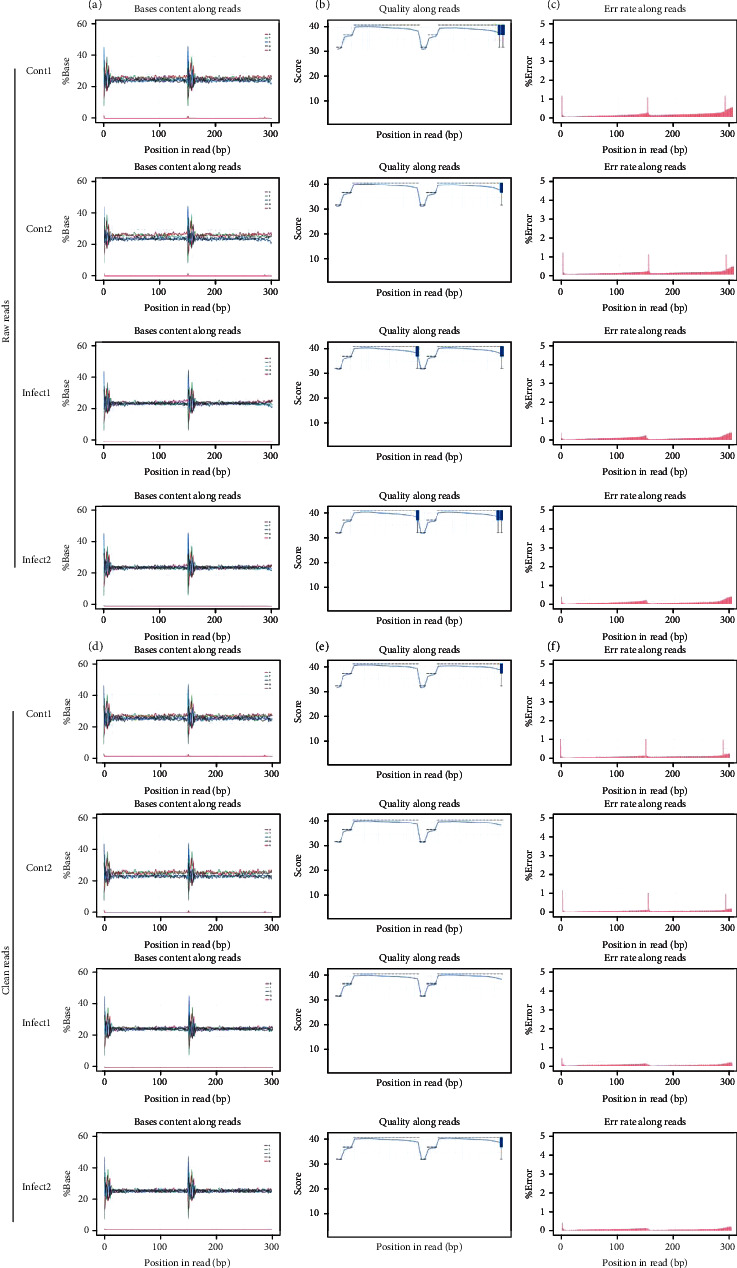
Sequencing data quality control. (a–c) Raw data quality control. (a) Composition of bases in each cycle. (b) The base quality distribution box plot. (c) Base error rate in raw reads. (d–f) Clean data quality control. (d) Composition of bases in each cycle. (e) The base quality distribution box plot. (f) Base error rate in raw reads.

In the current study, a total of 4 cDNA libraries were constructed by isolating total RNA from exosomes derived from *S. aureus*-infected and noninfected MAC-T cells. The raw reads in the cDNA library of different samples were cont1, 67572136; cont2, 66474707; infect1, 69033293; and infect2, 70292999. GC content (%) percentages were found to be cont1, 48.61; cont2, 47.63; infect1, 49.75; and infect2, 49.98 (Table 2). We conducted quality control analysis on raw data, and the results are as follows. The quality of the bases in raw reads was counted in each sequencing cycle. The base composition of each cycle was shown in Figure 2(a). As shown in Figure 2(b), the blue line represented the average base quality of the cycle in the base quality distribution box plot. Base error rate is limited to 0.11-0.13 in raw reads (Figure 2(c)). We filtered raw data to get clean data, and clean reads, GC%, Q20, Q30, and clean bases were counted in Table 3, and the quality control results of clean reads were shown in Figures 2(d)–2(f). In addition, when the clean reads were aligned with the bovine reference genome using HISAT2 software by an improved BWT algorithm (FM index), it was determined that the Total mapped reads or fragments belonging to all the samples were above 80% and was mapped in the reference genome (Table 4).

### 3.3. Distribution of Reads in the Chromosomes and Known Types of RNAs

**Figure 3:**
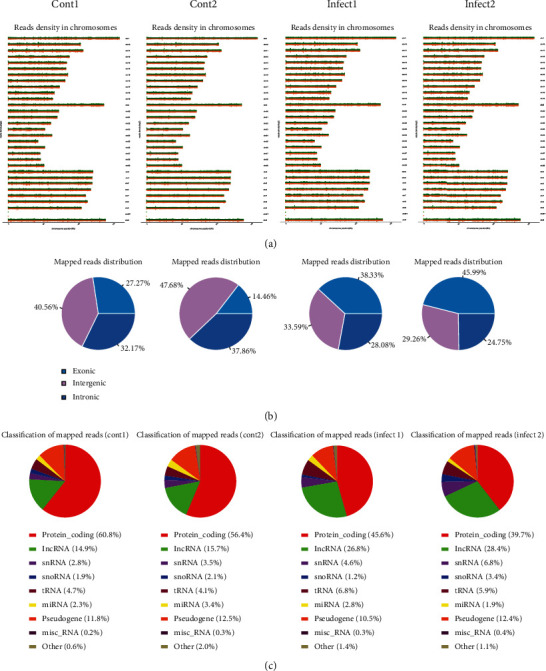
Mapped reads in the reference genome. (a) Distribution of reads in chromosome. (b) Location of reads in exonic, intronic, and intergenic. (c) RNA types of reads.

After mapped in the reference genome, we counted the position information of the genomes corresponding to all reads to evaluate the depth of sequencing data coverage. The distribution of reads on each chromosome was displayed in Figure 3(a). Subsequently, the reads aligned on the chromosome were annotated to exonic, intronic, and intergenic. Reads compared to the genome counted the distribution (Figure 3(b)). And the read distributions in the known RNA types were shown in Figure 3(c).

### 3.4. Differentially Expressed Gene Analysis in Exosomes Derived from *S. aureus*-Infected and Noninfected MAC-T Cells

**Figure 4:**
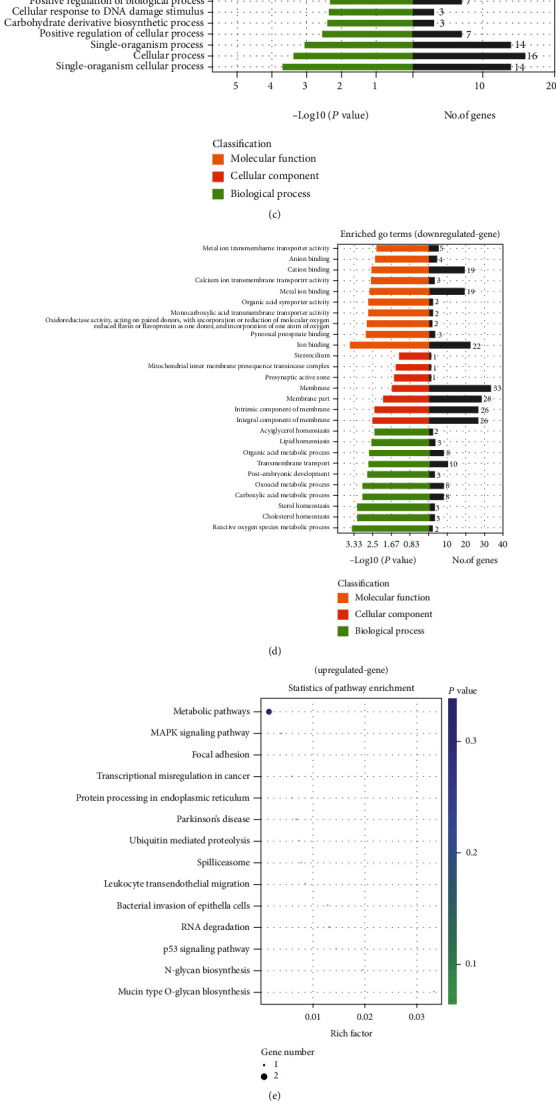
Differentially expressed genes analysis. (a) Volcano plot of differential expression gene. Red color represents genes with higher expression; green color represents genes with lower expression. (b) Clustering of differential expressed genes. Hierarchical clustering based on FPKMs, where log10 (FPKM+1) is used for clustering. Red color represents genes with higher expression, while blue color represents genes with lower expression. (c) GO enrichment analysis on upregulated genes. (d) GO enrichment analysis on downregulated genes. (e) The KEGG pathway enrichment analysis on upregulated genes. (f) The KEGG pathway enrichment analysis on downregulated genes.

DESeq2 was used to screen differentially expressed genes. We selected the differentially expressed genes between samples through the two levels of multiple of difference (∣log2 (Fold Change) | >1) and significance level (*Q* value < 0.05) [16–18]. The statistics of the number of differentially expressed genes in exosomes derived from *S. aureus*-infected and noninfected MAC-T cells were shown in Figure 4(a) and Table 5. There were a total of 186 gene expression disorders, of which 31 genes were significantly upregulated and 155 genes were significantly downregulated. We used the RPKM/COUNT of the differential gene as the expression level. Hierarchical clustering analysis was performed to cluster genes with the same or similar expression patterns into clusters and used different colored regions to represent different clustering grouping information to determine clustering mode of different sample control modes [15, 19]. In this study, the hierarchical clustering analysis of differentially expressed genes is shown in Figure 4(b).

GO enrichment analyses were conducted to search for the biological processes, cellular components, and molecular functions of differentially expressed genes in more detail [15, 20]. GO analysis was performed on the upregulated and downregulated genes. According to the enrichment point, all disordered genes are classified according to GO terms (Figures 4(c) and 4(d)). Moreover, KEGG pathway analysis results were calculated according to the enrichment points, and the best gene pathways related to the upregulated and downregulated genes were listed in Figures 4(e) and 4(f) and Table 5. These differentially expressed genes were involved in oxidative stress (CAMK4, CYP1A2, MLKL, NDUFA6, etc.), inflammation (FAS, ATM, IL1A, TNFRSF13C, BOLA-DOA, etc.), and apoptosis (ATM, FAS, SERPINB5, GORAB, etc.) processes.

### 3.5. Differentially Expressed mRNA and lncRNA Analysis in Exosomes Derived from *S. aureus*-Infected and Noninfected MAC-T Cells

**Figure 5:**
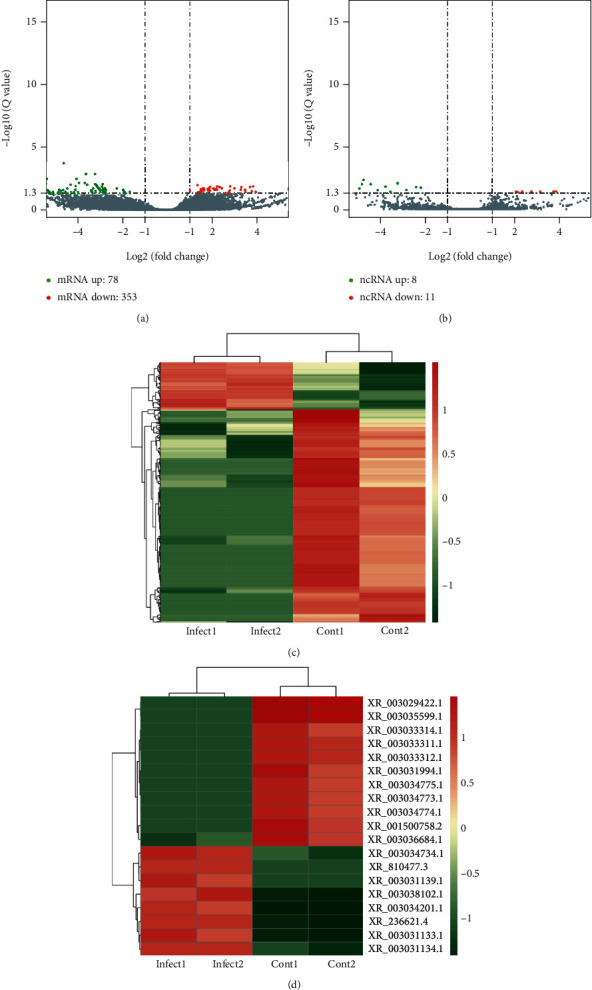
Differentially expressed mRNAs and lncRNAs. (a) Volcano plot of differential expression mRNA transcripts. (b) Volcano plot of differential expression lncRNA transcripts. Red color represents genes with higher expression; green color represents genes with lower expression. (c) Clustering of differential expressed mRNA. (d) Clustering of differential expressed lncRNA. Hierarchical clustering based on FPKMs, where log10 (FPKM+1) is used for clustering. Red color represents genes with higher expression, while blue color represents genes with lower expression.

The expression level of the transcript was calculated using the RPKM value. We found that there were 78 upregulated and 353 downregulated known mRNAs and 8 upregulated and 11 downregulated lncRNAs (Figures 5(a) and 5(b)). Upregulated lncRNAs were XR_003034773.1, XR_810477.3, XR_003031139.1, XR_003038102.1, XR_003034201.1, XR_236621.4, XR_003031133.1, and XR_003031134.1; downregulated lncRNAs were XR_003034734.1, XR_003033312.1, XR_003033314.1, XR_003034774.1, XR_003035599.1, XR_003033311.1, XR_001500758.2, XR_003029422.1, XR_003034775.1, XR_003031994.1, and XR_003036684.1. In addition, the differentially expressed mRNAs and lncRNAs were also clustered in the hierarchical cluster analysis map (Figures 5(c) and 5(d)).

### 3.6. Prediction of lncRNA Target Genes and mRNAs

**Figure 6:**
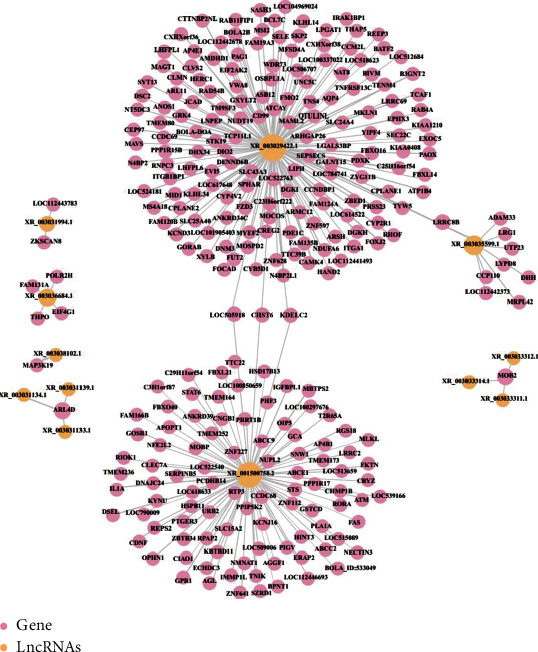
Network diagram of the interaction between lncRNA and target genes.

The mode of action of lncRNA regulating target genes could be divided into two categories: cis regulation (In Cis) and transregulation (In Trans). We made predictions about how the differentially expressed lncRNAs regulated the target genes. Differentially expressed lncRNAs were selected to draw a network diagram of the interaction between these lncRNAs and their target genes (Figure 6), so as to provide reference and help for the overall analysis of the functions of lncRNAs in samples. As illustrated in Figure 6, XR_001500758.2 targeted FAS, ATM, and IL1A; XR_003029422.1 targeted BOLA-DOA, TNFRSF13C, CAMK4, CYP2R1, SKP2, and NDUFA6.

### 3.7. GO Enrichment and KEGG Enrichment Analysis of lncRNA Target Genes and mRNAs

**Figure 7:**
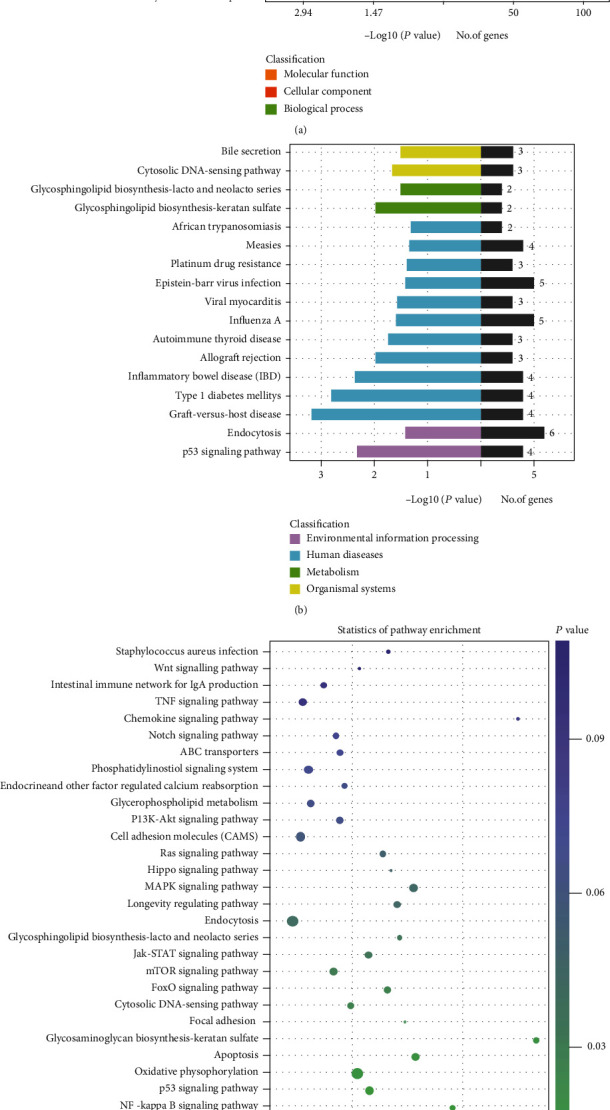
GO enrichment and KEGG enrichment analysis of lncRNA target genes and mRNAs. (a) GO enrichment analysis on lncRNA target genes. (b) lncRNA target gene KEGG enrichment on environmental information processing, human diseases, metabolism, and biological systems. (c) The KEGG pathway enrichment analysis on lncRNA target genes.

GO analysis was performed on the target gene and took the *P* < 0.05 of GO annotation enrichment as the significance threshold. GO terms of biological processes, cellular components, and molecular functions were shown 10 GO terms, respectively (Figure 7(a)). KEGG analysis was performed on the target gene and used the *P* < 0.05 of the enrichment degree of KEGG pathway as the significance threshold to obtain the analysis result. KEGG-enriched target gene pathways were classified according to environmental information processing, human diseases, metabolism, and biological systems. It was found that lncRNA target genes were most closely related to human diseases (Figure 7(b)). KEGG pathway analysis results are shown in the bubble diagram of the KEGG pathway (Figure 7(c)). Multiple target genes in the KEGG enrichment were involved in oxidative stress (FoxO signaling pathway (ATM, SKP2); longevity regulating pathway, cAMP signaling pathway, and calcium signaling pathway (CAMK4); reactive oxygen species metabolic pathways (CYP1A2); oxidative phosphorylation (NDUFA6)) and inflammation (TNF signaling pathway (FAS, MLKL); cytokine-cytokine receptor interaction (FAS, IL1A, and TNFRSF13C); chemokine signaling pathway (GRK4); MAPK signaling pathway (FAS, IL1A); and NF-kappa B signaling pathway (ATM, TNFRSF13C)) and apoptosis (p53 signaling pathway (ATM, FAS, SERPINB5, GORAB); apoptosis (ATM, FAS)).

### 3.8. Results of RT-qPCR Validation Was in accordance with RNA-Seq

**Figure 8:**
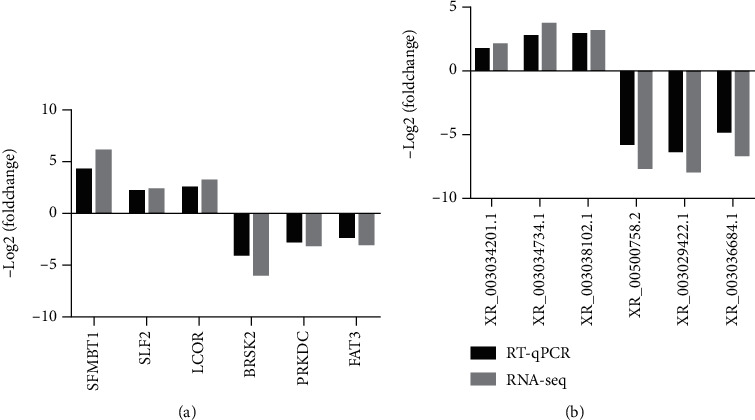
Comparison of relative expression of six selected differentially expressed mRNAs and lncRNAs between RT-qPCR and lncRNA-seq. (a) The relative expression level of selected mRNAs and lncRNAs was measured by RT-qPCR. (b) The relative expression levels of selected lncRNAs were measured by RT-qPCR.

6 differentially expressed mRNAs (BRSK2, PRKDC, FAT3, SFMBT1, SLF2, and LCOR) and 6 differentially expressed lncRNAs (XR_001500758.2, XR_003029422.1, XR_003036684.1, XR_003034201.1, XR_003034734.1, and XR_003038102.1) were chosen to verify the lncRNA-seq results. The results of qPCR were found to be in accordance with RNA-seq (Figures 8(a) and 8(b)), suggesting that RNA-seq results were reliable.

## 4. Discussion

Mastitis is an inflammation of the udders of dairy cows that is almost always caused by pathogenic microorganisms [21]. It seriously harms dairy farming and the dairy industry. Cow mammary epithelial cells can produce exosomes and excrete with milk during lactation, and exosomes can also communicate between cells through paracrine pathways. The cargo carried in the exosomes is the switch that initiates communication. The aim of this study was to investigate the expression of mRNA and lncRNA in exosomes derived from bovine mammary epithelial cells infected by *S. aureus*.

In this study, we used ultracentrifugation to separate the exosomes in the MAC-T cell culture medium. We directly observed saucer-shaped vesicles through transmission electron microscopy. The NTA instrument detected 80% of the extracellular vesicles with a size of 40-150 nm. In addition, the positive rates of CD81 and CD63, which are characteristic markers on the surface of exosomes, were more than 80% by flow cytometry. All the above data implied that exosomes were successfully isolated.

After the total exosomal RNA extracted, high-throughput sequencing was performed on the Illumina platform to reveal the lncRNA and gene expression profiles in the exosomes derived from *S. aureus*-infected and noninfected MAC-T cells. It was found that186 differentially expressed genes, 431 differentially expressed mRNAs, and 19 differentially expressed lncRNAs were screened out. Through GO function enrichment, most of the differentially expressed genes were involved in reactive oxygen species metabolic processes, signal transduction of gene expression regulation, Wnt signaling pathway, planar cell polarity pathway, regulation of macrophage activation, and various biochemical metabolic processes (carboxylic acid, oxoacid and lactate metabolic process, ketone and steroid catabolic process, etc.). In the enrichment of the KEGG pathway, the differentially expressed genes participated in the signal pathways were mainly concentrated in three aspects, including pathogen invasion and adhesion (bacterial invasion of epithelial cells, cell adhesion molecules, and tight junctions), metabolism (oxidative phosphorylation, reactive oxygen species metabolism), inflammation, and apoptosis (p53 signaling pathway, Ras signaling pathway, and MAPK signaling pathway). In addition to differentially expressed genes, the function of most of these lncRNAs was unclear. We hypothesized that lncRNA regulated the expression of these genes, so we predicted the function of lncRNA based on its closely related coding genes [22]. In this study, we estimated the target genes of differentially expressed lncRNA using cis and trans, then GO and KEGG analyses were performed on the target gene to find the potential role of lncRNA [15]. We found that these differentially expressed target genes were mainly related to inflammation or apoptosis signaling pathways through enrichment analysis of GO and KEGG pathways and coding-noncoding coexpression network analysis.

Differentially expressed lncRNA XR_001500758.2 and XR_003029422.1 were enriched in TNF signaling pathway, Notch signaling pathway, p53 signaling pathway, RIG-I-like receptor signaling pathway, NF-kappa B signaling pathway, MAPK signaling pathway, and PI3K-Akt signaling pathway. These signaling pathways are closely related to inflammation or apoptosis [23]. The target genes predicted for XR_001500758.2 included ATM, FAS, SERPINB5, PPIP5K2, SNW1, MLKL, IL1A, and PLA1A. ATM protein kinase is a product encoded by the telangiectasia ataxia-mutated gene ATM, which senses DNA damage, transmits DNA damage signals to downstream target genes, initiates stress systems, and produces cycle arrest, cell repair, and apoptosis [24, 25]. FAS, adapter proteins associated with the death domain (FADD and TRADD), belongs to TNFR family. FAS could activate NF-kappa B, MAPK, and PI3K-Akt signaling pathway to initiate transcription of inflammatory genes [26]. In addition, the target genes predicted for XR_003029422.1 included CAMK4, CYP2R1, SKP2, NDUFA6, BOLA-DOA, TNFRSF13C, GORAB, and ITGA1. CAMK4 was involved in the longevity regulating pathway, cAMP signaling pathway, and calcium signaling pathway in KEEG pathway enrichment. Previous studies have confirmed that the CAMK4 gene was closely related to human longevity and hypertension and regulated the immune response by activating transcription factors to regulate gene expression in immune cells [27, 28]. CYP1A2 was enriched in reactive oxygen species metabolic pathways. It has been reported that CYP1A2 could regulate various metabolic processes to participate in the production of reactive oxygen species during oxidative stress [29, 30]. SKP2 helps to inhibit oxidative stress induced-DNA damage and cell apoptosis, which limits the misincorporation of ROS-damaged dNTPs into genomic DNA, making cells more resistant to such stress [31]. NDUFA6 is a mitochondrial protein involved in mitochondrial energy metabolism. The dysregulation of NDUFA6 leads to mitochondrial dysfunction and oxidative stress [32]. Based on predictions, we found that lncRNA had multiple targets and was involved in the complex process of regulating the immune response.

In conclusion, the molecular mechanism of *S. aureus* infecting dairy cow mammary epithelial cells involves the paracrine mode of exosomal cargo lncRNA playing a regulatory role in pathogen invasion and adhesion, oxidative stress, inflammation, and apoptosis. The data obtained in this study would also provide valuable resources for understanding the lncRNA information in exosomes derived from dairy cow mammary epithelial cells, and conduced to the study of *S. aureus* infection in dairy cow mammary glands.

## Figures and Tables

**Table 1:** The sequence of primer used in this study.

Name	Primer sequence (5′-3′)	GenBank accession number	Product size (bp)
SFMBT1	Sense: CTAACCTCTTCGGTCCACG Antisense: CTTCACTTAGGGAATCGTCA	XM_024982617.1	315
SLF2	Sense: GAGACTAAGATACCGAAACC Antisense: GGAATACATGGCACAACT	NM_001102259.1	404
LCOR	Sense: GTATTCTTGAAGGGCTGTTTG Antisense: TGATCTCACGGGCCACTAG	XM_024985881.1	321
BRSK2	Sense: GCCTCACGCTAGAGCACATTC Antisense: GGCGGGTCTATCTCGTTCC	XM_024987615.1	310
PRKDC	Sense: CCTCCTGTTTGACCTTTG Antisense: TGACACTTCCCAGTTACTCC	NM_001256559.1	269
FAT3	Sense: GTATGAGAACTCGGCAGCAA Antisense: AATCCTCGCCTCGGACA	NM_001206882.1	257
LOC112446130	Sense: CCAGGGCGAGGCTTATC Antisense: AATTATGCAGTCGAGTTTCC	XR_003034201.1	82
CFAP54	Sense: GCGGGATGCCTGGTTAC Antisense: GTGGGAGACACGTTTGGTGA	XR_003034773.1	133
CCNT2	Sense: ACATCTCAGCACCCTCG Antisense: CCAGCCATTCACCCTAA	XR_003038102.1	433
LOC101905593	Sense: AGAAGGCAATGGCACCC Antisense: GACGGCAGTCCAACAGG	XR_001500758.2	218
LOC112441605	Sense: TGTTGTTGTTCCGTTGGTC Antisense: TCAGTGTTCGGTTATTTGC	XR_003029422.1	118
CLCN2	Sense: CCCGTCGTCCTCATCACTTT Antisense: TGCTTGCGATATGCACAAAA	XR_003036684.1	220

**Table 2:** Statistics of raw data quality.

Sample name	Total reads	Total reads (bp)	GC%	Error rate (%)	Q20 (%)	Q30 (%)
cont1	67572136	10135820400	48.61	0.11	98.64	96.29
cont2	66474707	9971206050	47.63	0.11	98.75	96.62
infect1	69033293	9854993950	49.75	0.13	98.26	95.53
infect2	70292999	10543949850	49.98	0.13	98.33	95.57

Total reads: statistics of total reads, each adjacent four lines contains the information of one read, and the total read number of each file is calculated; total base: the number of all bases in total data; clean reads: filter the original data, remove the linker sequence, remove the contaminated part, and remove the sequence containing more low-quality bases to obtain clean reads; GC%: the percentage of G and C in all bases; error rate: the error rate of sequencing; Q20, Q30: the percentage of total number of bases where the Phred score is greater than 20 and 30 which indicates base call accuracy.

**Table 3:** Summary of filter data.

Sample name	Raw reads	Raw bases	Clean reads	GC%	Q20 (%)	Q30 (%)	Clean bases	Clean bases%
cont1	67572136	10135820400	65650056	48.57	99.16	97.32	9689017234	95.59
cont2	66474707	9971206050	65006370	47.54	99.22	97.52	9514596643	95.42
infect1	69033293	9854993950	65789887	49.67	98.95	96.74	9383925239	95.22
infect2	70292999	10543949850	68826092	49.91	98.94	96.69	10102208096	95.81

Raw reads: statistics of raw reads, each adjacent four lines contains the information of one read, and the total read number of each file is calculated; raw base: the number of all bases in raw data; clean reads: filter the original data, remove the linker sequence, remove the contaminated part, and remove the sequence containing more low-quality bases to obtain clean reads; GC%: the percentage of G and C in all bases; Q20, Q30: the percentage of total number of bases where the Phred score is greater than 20 and 30 which indicates base call accuracy; clean bases: the number and length of sequences in clean reads calculate the number of bases; clean bases%: percentage of clean bases/raw base.

**Table 4:** Summary of reads mapped to reference genome.

Sample name	cont1	cont2	infect1	infect2
The effective reads	112941906 (100%)	123138246 (100%)	86025798 (100%)	107088594 (100%)
Total mapped	5194592 (4.60%)	3683147 (2.99%)	4047510 (4.70%)	3269932 (3.05%)
Multiple mapped	550374 (0.49%)	253166 (0.21%)	492395 (0.57%)	411581 (0.38%)
Uniquely mapped	4644218 (4.11%)	3429981 (2.79%)	3555115 (4.13%)	2858351 (2.67%)
Read1 mapped	2600145 (2.30%)	1843109 (1.50%)	2022180 (2.35%)	1633528 (1.53%)
Read2 mapped	2594447 (2.30%)	1840038 (1.49%)	2,025,330 (2.35%)	1636404 (1.53%)
Reads map to “+”	2,599,952 (2.30%)	1842749 (1.50%)	2025784 (2.35%)	1636402 (1.53%)
Reads map to “-”	2594640 (2.30%)	1840398 (1.49%)	2,021,726 (2.35%)	1633530 (1.53%)
Reads mapped in proper pairs	4815452 (4.26%)	3470648 (2.82%)	3800530 (4.42%)	3059418 (2.86%)

The effective reads: the remaining number of clean reads after removing rRNA reads will be used for subsequent genome alignment; total mapped: the number of sequencing sequences that can be aligned on the genome; multiple mapped: the number of sequencing sequences with multiple alignment positions on the sequence of reference; uniquely mapped: the number of sequencing sequences with unique alignment positions on the reference sequence; read-1, read-2 mapped: pair-end sequence, whose two parts that can be located on the number of genome, respectively; the statistical ratio of the two parts should be roughly the same; reads map to “+”: the number of reads aligned to the positive strand of the genome; reads map to “-”: the number of reads aligned to the genome on the negative strand; reads mapped in proper pairs: the relative distance of the pair end sequence mapping to the genome conforms to the length distribution of the sequenced fragments.

**Table 5:** Differentially expressed genes in exosomes and KEGG enrichment.

Gene		log2 (Fold_change)	*P* value	KEGG pathway
Up	ALG10	5.91	3.24*E*-04	N-Glycan biosynthesis; metabolic pathways
	UBE2G2	5.64	2.95*E*-05	Ubiquitin-mediated proteolysis; protein processing in endoplasmic reticulum
	ZMAT3	3.49	1.30*E*-04	p53 signaling pathway
	CCNT2	3.04	1.72*E*-05	Transcriptional misregulation in cancer
	C1D	2.28	5.08*E*-04	RNA degradation
	ARHGAP5	2.20	1.67*E*-04	Leukocyte transendothelial migration; focal adhesion
	TAOK1	1.92	3.38*E*-04	MAPK signaling pathway
	GALNT1	1.92	6.46*E*-05	Mucin type O-glycan biosynthesis; metabolic pathways
	CD2AP	1.55	2.41*E*-04	Bacterial invasion of epithelial cells
	HNRNPM	1.40	3.13*E*-04	Spliceosome

Down	CSF1	-8.66	7.76*E*-07	Ras signaling pathway; Rap1 signaling pathway; TNF signaling pathway; PI3K-Akt signaling pathway; cytokine-cytokine receptor interaction
	DSG1	-8.33	6.55*E*-06	Staphylococcus aureus infection
	NTNG2	-7.80	1.97*E*-04	Cell adhesion molecules; axon guidance
	NR1H3	-7.06	1.02*E*-04	PPAR signaling pathway; insulin resistance; hepatitis C; nonalcoholic fatty liver disease
	CYP1A2	-6.63	5.54*E*-04	Linoleic acid metabolism; tryptophan metabolism; retinol metabolism; steroid hormone biosynthesis; drug metabolism- cytochrome P450; metabolic pathways; chemical carcinogenesis
	MECOM	-5.36	1.44*E*-04	MAPK signaling pathway; chronic myeloid leukemia; pathways in cancer
	RELN	-4.79	1.39*E*-04	ECM-receptor interaction; PI3K-Akt signaling pathway; focal adhesion
	PLA2G4B	-4.23	3.12*E*-04	Linoleic acid metabolism; Glutamatergic synapse; Ras signaling pathway; Arachidonic acid metabolism; alpha-Linolenic acid metabolism; ether lipid metabolism; VEGF signaling pathway; fc epsilon RI signaling pathway; MAPK signaling pathway; GnRH signaling pathway; inflammatory mediator regulation of TRP channels; phospholipase D signaling pathway; oxytocin signaling pathway
	GRIN2A	-3.58	5.94*E*-04	cAMP signaling pathway; glutamatergic synapse; Ras signaling pathway; calcium signaling pathway; Rap1 signaling pathway
	PRKDC	-3.03	1.28*E*-04	Cell cycle; nonhomologous end-joining

## Data Availability

The data that support the findings of this study are available from the corresponding author upon reasonable request.

## Abbreviations

BMEC: Bovine mammary epithelial cells
LncRNA: Long noncoding RNA
*S. aureus*: *Staphylococcus aureus*
NTA: Nanoparticle tracking analysis
KEGG: Kyoto Encyclopedia of Genes and Genomes
GO: Gene Ontology
ATM: Ataxia-telangiectasia-mutated gene
MAPK: Mitogen-activated protein kinases
CAMK4: Calcium/calmodulin-dependent protein kinase 4
CYP1A2: Cytochrome P450 1A2
SKP2: S phase kinase-associated protein 2
NDUFA6: NADH dehydrogenase [ubiquinone] 1 alpha subcomplex subunit 6.
